# Antimicrobial Resistance Profile of *mcr-1* Positive Clinical Isolates of *Escherichia coli* in China From 2013 to 2016

**DOI:** 10.3389/fmicb.2018.02514

**Published:** 2018-10-23

**Authors:** Baiyuan Li, Bixia Ke, Xuanyu Zhao, Yunxue Guo, Weiquan Wang, Xiaoxue Wang, Honghui Zhu

**Affiliations:** ^1^State Key Laboratory of Applied Microbiology Southern China, Guangdong Provincial Key Laboratory of Microbial Culture Collection and Application, Guangdong Microbial Culture Collection Center (GDMCC), Guangdong Open Laboratory of Applied Microbiology, Guangdong Institute of Microbiology, Guangzhou, China; ^2^CAS Key Laboratory of Tropical Marine Bio-resources and Ecology, Guangdong Key Laboratory of Marine Materia Medica, RNAM Center for Marine Microbiology, South China Sea Institute of Oceanology, Chinese Academy of Sciences, Guangzhou, China; ^3^Center for Disease Control and Prevention of Guangdong Province, Guangzhou, China; ^4^University of Chinese Academy of Sciences, Beijing, China

**Keywords:** multidrug-resistant, clinical isolates, *Escherichia coli*, *mcr-1*, plasmid

## Abstract

Multidrug-resistant (MDR) *Escherichia coli* poses a great challenge for public health in recent decades. Polymyxins have been reconsidered as a valuable therapeutic option for the treatment of infections caused by MDR *E. coli.* A plasmid-encoded colistin resistance gene *mcr-1* encoding phosphoethanolamine transferase has been recently described in Enterobacteriaceae. In this study, a total of 123 *E*. *coli* isolates obtained from patients with diarrheal diseases in China were used for the genetic analysis of colistin resistance in clinical isolates. Antimicrobial resistance profile of polymyxin B (PB) and 11 commonly used antimicrobial agents were determined. Among the 123 *E. coli* isolates, 9 isolates (7.3%) were resistant to PB and PCR screening showed that seven (5.7%) isolates carried the *mcr-1* gene. A hybrid sequencing analysis using single-molecule, real-time (SMRT) sequencing and Illumina sequencing was then performed to resolve the genomes of the seven *mcr-1* positive isolates. These seven isolates harbored multiple plasmids and are MDR, with six isolates carrying one *mcr-1* positive plasmid and one isolate (14EC033) carrying two *mcr-1* positive plasmids. These eight *mcr-1* positive plasmids belonged to the IncX4, IncI2, and IncP1 types. In addition, the *mcr-1* gene was the solo antibiotic resistance gene identified in the *mcr-1* positive plasmids, while the rest of the antibiotic resistance genes were mostly clustered into one or two plasmids. Interestingly, one *mcr-1* positive isolate (14EC047) was susceptible to PB, and we showed that the activity of MCR-1-mediated colistin resistance was not phenotypically expressed in 14EC047 host strain. Furthermore, three isolates exhibited resistance to PB but did not carry previously reported *mcr*-related genes. Multilocus sequence typing (MLST) showed that these *mcr-1* positive *E*. *coli* isolates belonged to five different STs, and three isolates belonged to ST301 which carried multiple virulence factors related to diarrhea. Additionally, the *mcr-1* positive isolates were all susceptible to imipenem (IMP), suggesting that IMP could be used to treat infection caused by *mcr-1* positive *E. coli* isolates. Collectively, this study showed a high occurrence of *mcr-1* positive plasmids in patients with diarrheal diseases of Guangzhou in China and the abolishment of the MCR-1 mediated colistin resistance in one *E. coli* isolate.

## Introduction

*Escherichia coli* is an important member of the intestinal microbiota of humans and animals (Finegold et al., 1983). Most *E*. *coli* strains are harmless and even benefit the host by preventing colonization of the harmful pathogens (Chang et al., 2004). However, some *E*. *coli* strains have evolved into pathogenic *E*. *coli* by the acquisition of virulence factors through plasmids, transposons, bacteriophages, and/or pathogenicity islands (Nataro and Kaper, 1998). Pathogenic *E*. *coli* has been recognized as an important cause of extraintestinal and intestinal infections in both humans and companion animals (Russo and Johnson, 2003; Wiles et al., 2008). In addition, the emergence of *E*. *coli* strains showing resistance to broad-spectrum of antimicrobial agents had been reported in the 1980s (Novick, 1981). Emergence of multidrug-resistant (MDR) *E. coli* has become an urgent global health threat due to the lack of effective antimicrobial agents in recent decades.

Polymyxins (including colistin) have been reconsidered as a valuable therapeutic option for the treatment of infections caused by Gram-negative bacteria with MDR including *E*. *coli* (Biswas et al., 2012). In 2015, a plasmid-encoded colistin resistance gene *mcr-1* encoding phosphoethanolamine transferase was described in Enterobacteriaceae isolated from humans and livestock in China (Liu et al., 2016). Since then, plasmid-mediated polymyxin resistance by *mcr-1* has been reported worldwide in livestock, food, and humans (Poirel et al., 2017). Currently, eight types of *mcr* genes [*mcr-1* (1626 bp), *mcr-2* (1617 bp), *mcr-3* (1626 bp), *mcr-4* (1626 bp), *mcr-5* (1644 bp), *mcr-6* (1617 bp), *mcr-7* (1620 bp), and *mcr-8* (1698 bp)] have been described in *E. coli* and other Gram-negative bacteria (Liu et al., 2016; Xavier et al., 2016; AbuOun et al., 2017; Borowiak et al., 2017; Carattoli et al., 2017; Yin et al., 2017; Wang et al., 2018;Yang et al., 2018). Monitoring of colistin-resistance and MDR as well as determining the genetic source of the colistin-resistant and MDR in clinical isolates are thus needed in the clinical treatments of *E. coli*-related infections.

The purpose of this study was to determine the prevalence of polymyxin resistance and MDR among the 123 *E. coli* isolates obtained from patients with diarrheal diseases in China from 2013 to 2016. The profile of resistance to 12 commonly used antimicrobial agents including polymyxin B (PB) was analyzed in these isolates. In order to determine the distribution of the resistance genes in the *mcr-1* positive isolates, we sequenced and *de novo* assembled the chromosomes and the plasmids of the seven *mcr-1* positive isolates. Since a recent study showed the shufflon is one of the most difficult regions for *de novo* genome assembly especially for those of IncI2 plasmids carrying the *mcr-1* gene (Sekizuka et al., 2017), a hybrid sequencing analysis approach using the single-molecule, real-time (SMRT) sequencing and Illumina sequencing was performed to avoid the possible misassemble caused by these shufflons.

## Materials and Methods

### Bacterial Identification and PCR

In total, 123 *E*. *coli* isolates were collected from the fecal samples of patients with diarrheal diseases during 2013 to 2016 by the Guangdong Provincial Center for Disease Control and Prevention (CDC) in China. These isolates were subsequently identified as *E. coli* strains using PCR amplification of the 16S rRNA gene using the commonly used primer pair 27F/1492R (Weisburg et al., 1991) (Supplementary Table S1). The presence of the *mcr-1* gene in these isolates was screened via PCR using primers list in Supplementary Table S1 as described previously (Liu et al., 2016; Xavier et al., 2016). The presence of *mcr-1*-*pap2* region in the *rhmT* gene was confirmed by PCR with the primers RhmT-F and RhmT-R (Supplementary Table S1).

### Susceptibility Testing

The antibiotics tested were ampicillin (AMP), PB, cefoxitin (CFX), ceftazidime (CAZ), imipenem (IMP), cefotaxime (CTX), cefepime (FEP), ciprofloxacin (CIP), gentamycin (GEN), sulfamethoxazole–trimethoprim (SXT), chloramphenicol (CM), and tetracycline (TET) in this study (Supplementary Table S2). The antibiotic resistance level was described by the minimum inhibitory concentrations (MICs) determined using a custom-made 96-well MIC panel (Xingbo Biotech, Shanghai, China). Results were interpreted according to the criteria of the Clinical and Laboratory Standards Institute (CLSI) (Clinical and Laboratory Standards Institute, 2017).

### Whole-Genome Sequencing

Whole-genome sequencing of seven *mcr-1* positive isolates was performed by Shanghai Majorbio Bio-Pharm Technology Co., Ltd. using Illumina HiSeq 4000 sequencing technology with a 350-bp size library. Paired-end Illumina reads were assembled with SOAP denovo v2.04^1^. The gaps of seven *mcr-1* positive isolates were closed using a PacBio RS II system (Pacific Biosciences, Menlo Park, United States) with a 10-kb library and P6/C4 chemistry. *De novo* assembly was performed with HGAP v3 (Pacific Biosciences). The complete genome sequence was annotated using Glimmer 3.02^2^ and BLASTN. Multilocus sequence typing (MLST) profiles, serotyping, virulence factors, antibiotic resistance gene contents and plasmid incompatibility groups were analyzed through the website of the Center for Genomic Epidemiology^3^. The nucleotide sequences of the genomes and plasmids of seven *mcr-1* positive isolates have been submitted to GenBank with accession Nos. CP024127-CP024158 and listed in Table 1.

**Table 1:** Genomic features of seven *mcr-1* positive *E*. *coli* isolates.

Strains	Year	MLST^a^	Serotype	Chromosome/Plasmid	Inc Type	Size (bp)	Accession numbers	Virulence factors
14EC001	2014	ST793	O115:H10	Chromosome	–	5,072,975	CP024127	*gad*, *nleA*, *nleB*, *astA*, *iha*, *espJ*, *cif*, *iss*, *tir*, *espA*, *espF*
				p14EC001a	IncP1	50,013	CP024128	–
				p14EC001b	IncFIB	123,884	CP024129	*katP*, *etpD*
				p14EC001c	IncFIB/IncFIA	88,460	CP024130	–
14EC007	2014	ST301	O180:H2	Chromosome	–	5,084,741	CP024131	*gad*, *nleB*, *tccP*, *cif*, *espF*, *espA*, *eae*, *tir*
				p14EC007a	IncX4	35,098	CP024132	–
				p14EC007b	IncFII/IncR/IncFIB	190,293	CP024133	*katP*, *etpD*
14EC017	2014	ST301	O70:H2	Chromosome	–	5,199,281	CP024134	*gad*, *nleB*, *nleC*, *espF*, *espB*, *espA*, *eae*, *tir*
				p14EC017a	IncI2	63,978	CP024135	–
				p14EC017b	IncFII/IncX1	93,781	CP024136	–
				p14EC017c	IncFIB	107,279	CP024137	*cma*, *etpD*, *katP*, *nleA, mchF*
14EC020	2014	ST117	O24:H4	Chromosome	–	4,914,884	CP024138	*gad*, *iss*, *lpfA*, *ireA*, *hlyE*
				p14EC020a	IncI2	64,765	CP024139	–
				p14EC020b	IncFIB/IncFIC	166,233	CP024140	*iroN*, *iss*, *cma*
14EC029	2014	ST88	O8:H10	Chromosome	–	4,981,062	CP024141	*gad*, *lpfA*
				p14EC029a	IncI2	66,596	CP024142	–
				p14EC029b	IncN, IncHI2, IncHI2A	254,423	CP024143	–
				p14EC029c	IncFIA/IncFIB	96,973	CP024144	–
				p14EC029d	IncFIB/IncFIC	106,478	CP024145	*ltcA*, *astA*, *stb*
				p14EC029e	IncI1	88,553	CP024146	–
14EC033	2014	ST2064	O52:H45	Chromosome	–	4,639,454	CP024147	*gad*
				p14EC033a	IncI2	62,585	CP024148	–
				p14EC033b	IncX4	33,301	CP024149	–
				p14EC033c	Incl1	108,710	CP024150	–
				p14EC033d	IncFIC/IncFIB	97,858	CP024151	–
				p14EC033e	IncI1	87,351	CP024152	–
				p14EC033f	IncFIA/IncN/IncFIB	98,181	CP024153	–
				p14EC033g	ND	84,404	CP024154	–
14EC047	2014	ST301	O115:H2	Chromosome	–	5,060,393	CP024155	*gad*, *nleB*, *nleC*, *espF*, *espB*, *espA*, *eae*, *tir*
				p14EC047a	IncI2	60,258	CP024156	–
				p14EC047b	IncFII	88,736	CP024157	–
				p14EC047c	IncFIB	106,324	CP024158	*katP*, *cma*, *mchF*, *etpD, nleA*

^a^*Multilocus sequence typing. ND, no plasmid replicons detected. –, undetected*.

### Construction of Strains and Vectors

The pUC19 and pEX18Gm vectors were used to express *mcr-1* gene in *E*. *coli* K-12 or in 14EC047. The coding region and its promoter sequence of *mcr-1* genes were amplified with primer pair listed in Supplementary Table S1 with genomic DNAs from 14EC001 and 14EC047 as well as pHNSHP45 plasmid DNA as template, respectively. PCR products (pHNSHP45 plasmid DNA as template) were digested with BamHI and SalI and purified with using a gel extraction kit (Qiagen, Hilden, Germany). The purified PCR products were ligated into the pEX18Gm and transferred into *E*. *coli* K-12 and 14EC047 by electroporation. Furthermore, the *mcr-1* gene and their promoter sequence from 14EC001 and 14EC047 were ligated into a cloning vector pUC19 yielding pUC19-*mcr-1*. pUC19-*mcr-1* was then transferred into *E*. *coli* K-12 by electroporation. The correct constructs were verified by DNA sequencing.

## Results and Discussion

### Antimicrobial Resistance Profile of Clinical *E. coli* Isolates

**FIGURE 1:**
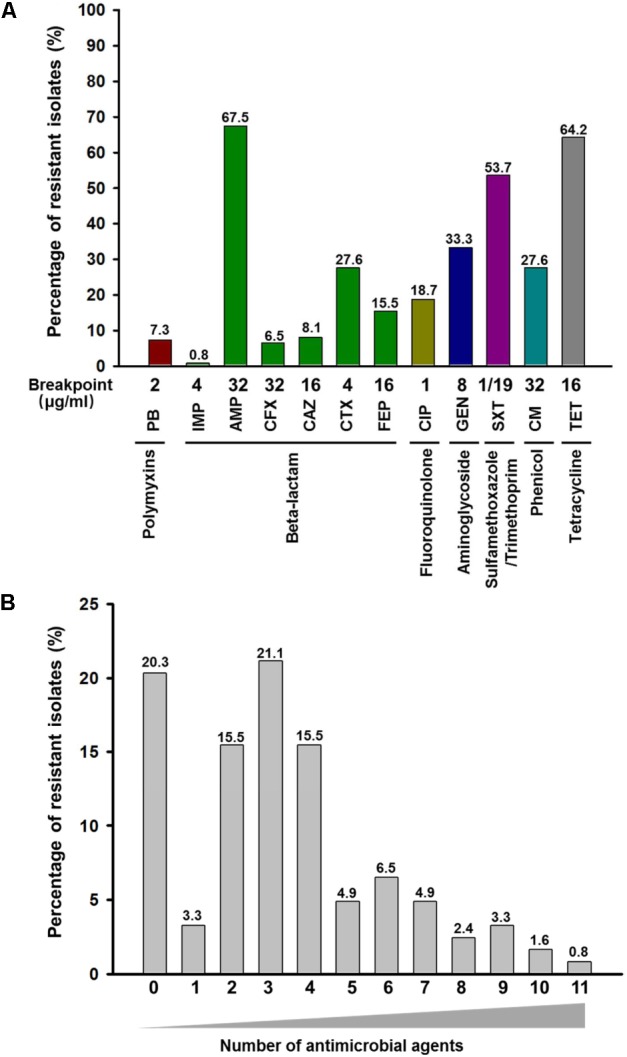
Frequency and distribution of resistance to antimicrobial agents among 123 *E*. *coli* isolates obtained from clinical specimens in China. **(A)** Frequency of resistance to each antimicrobial agent among 123 *E*. *coli* isolates. The number on top of each column represents the percentage of resistant isolates to each antimicrobial agent. PB, polymyxin B; IMP, imipenem; AMP, ampicillin; CFX, cefoxitin; CAZ, ceftazidime; CTX, cefotaxime; FEP, cefepime; CIP, ciprofloxacin; GEN, gentamycin; SXT, sulfamethoxazole-trimethoprim; CM, chloramphenicol; TET, tetracycline. **(B)** Occurrence of multidrug resistance among 123 *E*. *coli* isolates. The *x*-axis indicates the number of antimicrobial agents. The number on top of each column represents the percentage of resistant isolates to different numbers of antimicrobial agents.

The results of the susceptibility testing for all isolates are summarized in Figure 1. Most isolates studied (*n* = 98; 79.7%) were resistant to at least one antimicrobial agents, and one strain was resistance to 11 antimicrobial agents tested. Among these strains, resistance to three antimicrobial agents (21.1%) was most frequent, and the frequency of MDR *E*. *coli* isolates was 61.0% (Figure 1B and Supplementary Table S2). Similarly, a high proportion of MDR *E*. *coli* in human isolates was also described previously in the United States (Tadesse et al., 2012). These isolates were most frequently resistance to AMP (*n* = 83, 67.5%), SXT (*n* = 63, 53.7%), and TET (*n* = 79, 64.2%). These isolates were most susceptible to IMP and the resistance rate to IMP was only 0.8%. Furthermore, nine isolates (7.3%) had higher MIC values of PB (4 or 8 mg/L) than the rest of the isolates (MIC < 2.0 mg/L) (Supplementary Table S2). The prevalence of colistin resistance in clinical isolates found in this study is higher than the previous reports (usually lower than 1%) (Gales et al., 2011; Lu et al., 2018). In China, colistin has been widely used for the treatment of diarrhea in food-producing animals for decades, especially pigs and poultry. The transmission of colistin resistance gene *mcr-1* has been reported to be associated with the food chain (Zurfluh et al., 2017). Thus, the high prevalence of *mcr-1* in these diarrheal patients might be associated with food-producing animals. Our analysis showed that these nine colistin resistance strains were all MDR isolates, with resistance to at least three different antimicrobial agents. Nevertheless, eight out of the nine colistin resistance strains were susceptible to IMP, suggesting that IMP could be used to treat infection caused by colistin resistance *E*. *coli*.

The relatively low detection of IMP resistance in clinical *E. coli* isolates of Guangzhou (China) is consistent with previous report in Germany (Falgenhauer et al., 2016) and Vietnam (Hoang et al., 2017), respectively. However, the distribution of other types of resistance detected this study is different from the clinical *E. coli* isolates obtained in the United States (Karlowsky et al., 2002; Tadesse et al., 2012; Lob et al., 2016). It has been suggested that differences in resistance profile of geographically distinct regions maybe caused by the different use of antimicrobial agents in these regions (Gupta et al., 2001; Sannes et al., 2004).

### Prevalence and Genetic Source of Colistin Resistance in Clinical *E. coli* Isolates

We first used PCR screening to detect the presence of the *mcr-1* gene in these 123 isolates, and 7 isolates (5.7%) were positive for *mcr-1* (Supplementary Figure S1). Among the nine isolates with higher MIC values of PB, six of them were detected to have the *mcr-1* gene by PCR screening but three of them without the *mcr-1* gene. To screen other *mcr* genes among three colistin-resistant *mcr-1*-negative isolates, whole-genome sequencing was performed. Sequencing analysis revealed that these three colistin-resistant isolates harbored no *mcr* genes using sequences of the eight *mcr-1* related genes. Among the three isolates, strains of 14EC035 and 14EC043 exhibit similar MIC values of PB as the *mcr-1* positive strains. However, 14EC045 exhibits a much higher MIC value of PB (>32 mg/L) than the MCR-mediated colistin resistance. Sequencing of the *pmrA*, *pmrB*, *pmrC*, *pmrD*, *mgrB*, *phoP*, and *phoQ* was performed using whole genome sequencing and two chromosomal mutation in PhoQ [Glu to Asp at amino acid position 464 (E464D) and Ala to Thr at amino acid position 482 (A482T)] were detected in 14EC045. E464D and A482T mutations in *phoQ* previously observed by Delannoy et al. (2017) in colistin resistance *mcr-1* negative *E*. *coli* isolates. Thus, we suggested that two mutations in the chromosomal encoded gene *phoQ* might be responsible for the colistin resistance in *E*. *coli* 14EC045.

**FIGURE 2:**
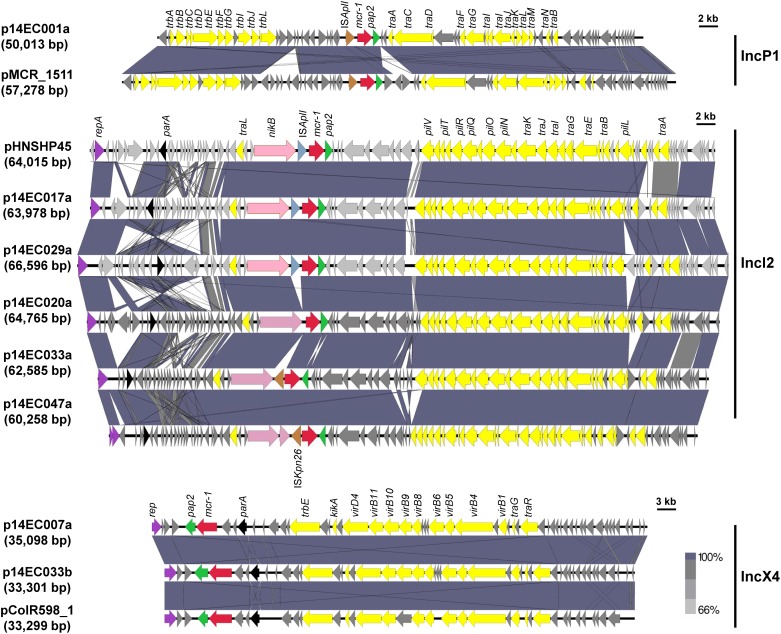
Sequence alignment of the eight *mcr-1* carrying plasmids and pMCR_1511, pHNSHP45, or pColR598_1. Arrows show the directions of putative open reading frames (ORFs), and the length of the arrow is proportional to the size of each ORF. The gene *mcr-1* is marked in red. IS*Apl1*, IS*Kpn26*, and *pap2* gene are marked in blue gray, brown, and green. Nickel transport system permease gene (*nikA*), replication initiation protein (*repA*), and partition protein (*parA*) are marked in pink, purple, and black. Conjugal transfer genes are marked in yellow. Regions of homology between sequences (>66%) are indicated by the graded shading.

To further determine the distribution of *mcr* genes and to avoid misassemble cause by shufflons, the genomes of these seven isolates were determined using a combination of Illumina HiSeq 4000 sequencing technology and SMRT (also known as PacBio RS II system) sequencing technology. The genome features of the seven *mcr-1* positive isolates are summarized in Table 1. Sequencing analysis revealed that these seven isolates all carried at least one copy of the *mcr-1* gene, and the *mcr-1* genes were most carried on plasmids, which are the primary vehicles for the dissemination of antibiotic resistance genes (Carattoli, 2013). Since the discovery of the *mcr-1* gene in China in 2015 (Liu et al., 2016), *mcr-1* positive strains have been found in different *Enterobacteriaceae* from various sources worldwide (Poirel et al., 2017). Furthermore, *mcr-1* has been found on plasmids representing a diverse range of incompatibility groups. In particular, *mcr-1* carrying plasmids belonging to the IncI2, IncHI2, and IncX4 families account for the majority of such sequences submitted to GenBank (Snesrud et al., 2016). In this study, a total of eight *mcr-1* carrying plasmids were recovered, with the isolate 14EC033 yielding two distinct *mcr-1* carrying plasmids. The eight *mcr-1* positive plasmids (30–70 kb in size) included five IncI2 type, two IncX4 type and one IncP1 type. The *mcr-1* genes on the eight plasmids shared 100% sequence identity with that of pHNSHP45 (Figure 2). A BLASTN comparison was carried out for the eight *mcr-1* carrying plasmids identified in this study and the corresponding plasmids reported previously (Figure 2). All of the Incl2-type plasmids share a high homology with pHNSHP45. The IncP1-type plasmid of p14EC001a shared high similarity (100% coverage and 99% identity) with the plasmid pMCR_1511 (KX377410.1), which was isolated from *Klebsiella pneumoniae* WCHKP1511 (Zhao et al., 2017). In contrast, the IncX4-type plasmids of p14EC007a and p14EC033b shared 99% identity with the plasmid pColR598_1 of *E. coli* ColR598 (MF175190.1), which was recently described in Switzerland (Zurfluh et al., 2017).

**FIGURE 3:**
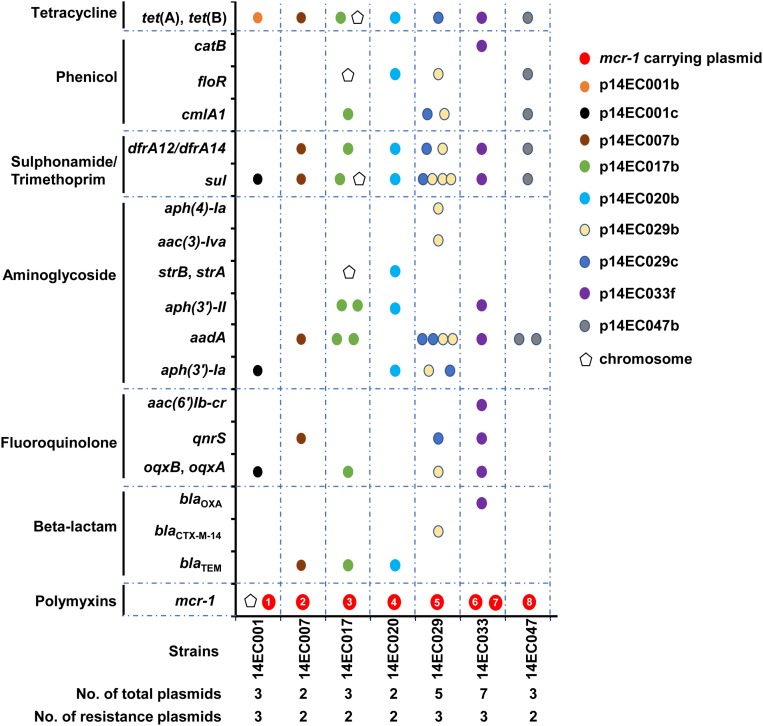
Distribution of the antimicrobial resistance gene is indicated by pentagon (chromosome) or circle (plasmid) in the seven *mcr-1* positive strains. The eight *mcr-1* carrying plasmids are indicated by red circles and are numbered as 1–8 for p14EC001a, p14EC007a, p14EC017a, p14EC020a, p14EC029a, p14EC033a, p14EC033b, and p14EC047a, respectively.

Moreover, isolate 14EC001 contains two copies of the *mcr-1* gene, with one on an IncP1-type plasmid (p14EC001a) and the other one on the host chromosome (Figure 3 and Supplementary Table S3). Comparative analysis revealed that the sequence of *mcr-1*-*pap2* region (2,600 bp in length) inserted in the host chromosome of 14EC001 is identical to a region in the plasmid p14EC001a of isolate 14EC001 (Supplementary Figure S2A). This insertion region contains one copy of the *mcr-1* gene and its downstream gene *pap2* and is inserted inside the *rhmT* gene in the host chromosome of 14EC001. Gene *rhmT* encodes a putative L-rhamnonate transporter which is a member of the major facilitator superfamily (MFS) and is likely to be in the *rhmRDTA* operon. MFS is one of the two largest families of membrane transporters found on Earth and ubiquitously distributed in bacteria, archaea, and eukarya (Reddy et al., 2012). Furthermore, *rhmT* and the *rhmRDTA* operon, without the *mcr-1*-*pap2* region, are highly conserved among *E*. *coli* strains including the commensal *E. coli* K-12 strain (Supplementary Figure S2A). PCRs using primers flanking the *rhmT* gene further confirmed the presence of an extra 2.6 kb region in the coding region of *rhmT* in strain 14EC001 but not in other *E. coli* strains (Supplementary Figure S2B). To investigate whether chromosome-encoded *mcr-1* is still functional, we amplified a 1983-bp DNA fragment containing the *mcr-1* gene and its 357 bp upstream region using the genomic DNA of 14EC001 as a template and cloned it into pUC19 (pUC19-*mcr-1* chromosome) (Supplementary Figure S2C); the resistance of the *E. coli* K-12 transformant to PB was determined. The results showed that the transformant with the *mcr-1* gene from the chromosome exhibited increased resistance to PB, with its MIC increasing from <0.5 mg/L (empty pUC19) to 4.0 mg/L (pUC19-*mcr-1* chromosome). The same MIC value of PB was obtained when a 2827-bp DNA fragment containing the *mcr-1* gene and its 1274 bp upstream region (including IS*Apl1* element) was amplified using p14EC001a as a template and cloned into pUC19 (pUC19-*mcr-1* plasmid) (Supplementary Figure S2C). Indeed, strain 14EC001 exhibited the highest MIC of PB (8 mg/L), while the MICs of the other the PB-resistant isolates were all 4 mg/L (Supplementary Table S2).

### One *mcr-1* Positive Isolate Does Not Express the Colistin-Resistance Phenotype

Unexpectedly, one *mcr-1* positive isolate, 14EC047, is susceptible to PB (MIC < 0.5 mg/L). Isolate 14EC047 harbored three different plasmids including one *mcr-1* charring plasmid. Genetic analysis showed that insertion elements were found in four *mcr-1* carrying plasmids. In plasmids of p14EC017a and p14EC029a, a single copy of IS*Apl1* is located upstream of *mcr-1*. In plasmids of p14EC047a and p14EC033a, a single copy of IS*Kpn26* is located upstream of *mcr-1* (Figure 2). To test whether the presence of the IS*Kpn26* affects *mcr-1* activity, we cloned the *mcr-1* gene and the promoter region containing IS*Kpn26* from 14EC047 into pUC19 vector to make pUC19-*mcr-1.* When electroplated into *E*. *coli* K-12 host, the transformant exhibited increased colistin resistance (MIC = 4.0 mg/L), suggesting that IS*Kpn26* does not affect the colistin resistance conferred by MCR-1 (Supplementary Figure S3). This is consistent with a recent report that the insertion of IS*Kpn26* to the upstream of *mcr-1* in *E*. *coli* isolates ZJ148 and ZJ1653 did not affect the colistin resistance (Shen et al., 2018). We then reasoned that the host strain 14EC047 might suppress the activity of MCR-1. Next, we cloned the *mcr-1* gene from pHNSHP45 into pEX18Gm vector to construct pEX18Gm-*mcr-1*. As expected, transformant *E*. *coli* K-12/pEX18Gm-*mcr-1* exhibited increased colistin resistance (MIC = 4.0 mg/L). In contrast, transformant 14EC047/pEX18Gm-*mcr-1* was susceptible to colistin (MIC < 0.5 mg/L) (Supplementary Figure S3). This result showed that the colistin resistance conferred by MCR-1 was abolished in 14EC047. Further investigation is needed to elucidate the underlying mechanism. Since high expression of *mcr-1* in *E. coli* could result in reduced growth rate and competitive ability during infection, *mcr-1* silencing might be a compensatory adaptation in pathogenic isolates (Yang et al., 2017).

### Distribution of the Virulence Factors in *mcr-1* Positive Isolates

The *E*. *coli* isolates analyzed in this study were recovered from diarrheal patients in China and diarrhea is one of the major clinical manifestations of *E*. *coli* (Gibbs et al., 2004). MLST analysis assigned these seven *mcr-1*-positive isolates to five distinct sequence types (STs), with three isolates belonging to ST301 and the rest of them belonging to ST793, ST117, ST88, and ST2064. ST117 and ST88 have been previously reported in *mcr-1* positive pathogenic *E*. *coli* (Maluta et al., 2014; Manges et al., 2015). However, ST301, ST793, and ST2064 were not reported previously in *mcr-1* positive *E*. *coli* isolates. ST10 are widely distributed in human diarrheagenic *E*. *coli* infection in Hangzhou of China (Yu et al., 2018), but in this study, we found ST301 was more common in *mcr-1* positive diarrheagenic *E*. *coli* infection in Guangzhou of China from year 2013–2016.

Next, the distribution of virulence factors were then analyzed through *in silico* analysis (see footnote 4) among the seven *mcr-1* positive isolates (Table 1). A total of 26 different virulence genes were identified among these *mcr-1* positive isolates, and all seven *mcr-1* positive isolates were positive for the *gad* gene which encodes protein involved in the glutamate decarboxylase (GAD) system. The GAD system is important for *E*. *coli* to survive in low pH conditions such as passage through the stomach after ingestion (Lin et al., 1996). Three isolates (14EC007, 14EC017, and 14EC047) belonging to ST301 all carried virulence genes including *nleB*, *espF*, *espA*, *eae*, *tir*, *katP*, and *etpD* besides *gad*. Previous studies have suggested that virulence factors such as *eae*, *nleA*, *nleB*, *nleC*, *lpfA* genes, which are associated with diarrhea were present in these isolates (Menard and Dubreuil, 2002; Afset et al., 2006). Two isolates (14EC001 belonging to ST793 and 14EC029 belonging to ST88) carried genes related to enterotoxin such as *astA*, *ltcA*, and *stb*. Enterotoxin is a major cause of diarrhea in infants in developing countries and in travelers to those regions (Gupta et al., 2008). Additionally, isolate 14EC020 of ST117 carried virulence factors including *gad*, *iss*, *lpfA*, *ireA*, *hlyE*, *iroN*, and *cma*. This study showed that *mcr-1* positive isolates possessed a broad range of virulence factors.

### Distribution of the Antimicrobial Resistance Genes in *mcr-1* Positive Isolates

Identification of resistance genes in the genome of the seven isolates was performed using ResFinder from the Center for Genomic Epidemiology (see footnote 3). In addition to the seven classes of antibiotic agents tested in the custom-made 96-well MIC panel, resistance genes related to rifampicin, MLS, and fosfomycin resistance were also determined. As shown in Figure 3, all isolates harbored multiple plasmids (up to seven plasmids), and all isolates harbored two to three plasmids with antibiotic resistance genes. Mobile genetic elements, particularly plasmids, are associated with the acquisition and dissemination of antimicrobial resistance genes in *E*. *coli* (Schroeder et al., 2004; Kadlec and Schwarz, 2008). When carried on transposons and plasmids, the resistance genes could be transmitted both intraspecies and interspecies (Galani et al., 2010; Huang et al., 2012). Our studies demonstrated that most of the resistance genes detected were carried on plasmids. In particular, except for *mcr-1* and *tet* (B) genes, many resistance genes were located within one single plasmid even in the strain harboring multiple plasmids. Moreover, all the *mcr-1* carrying plasmids only encode resistance to polymyxin. Isolates 14EC007 and 14EC020 each harbored two plasmids and showed resistance to six different classes of antimicrobial agents, with the *mcr-1* gene located on one plasmid and all of the rest resistance genes on the other plasmid. Isolates 14EC029 and 14EC033 harbored the highest number of plasmids and the highest number of resistance genes. These two isolates each harbored one IncI2 type *mcr-1* carrying plasmid. Additionally, the two plasmids in 14EC029 carried 9 and 15 resistance genes, respectively, and while no resistance gene was found in the rest of the two plasmids. Similarly, isolate 14EC033 harbored one IncX4 type *mcr-1* carrying plasmid and another plasmid carrying 11 resistance genes including AAR-3 gene conferring resistance to rifampicin. None of the resistance genes was identified in the rest of the four plasmids in 14EC033 (Figure 3 and Supplementary Table S3). One exception is plasmid p14EC001b in isolate 14EC001, which only carried *tet* (B) gene encoding TET resistance but the rest of the TET resistance genes were clustered with other resistance genes in other plasmids.

In terms of the distribution of the resistance genes in these isolates, in addition to resistance to polymyxin, all of these strains carry genes encoding resistance to aminoglycoside, SXT, and TET. Genes encoding various extended spectrum β-lactamases were identified in five (but not in 14EC001 and 14EC047) of the seven *mcr-1* positive isolates, exhibiting resistant to at least one antimicrobial agent within this class. Although six of seven *mcr-1* positive isolates were resistant to AMP, only one of these isolates showed resistance to other antibiotics tested of this class. Isolate 14EC029, which was positive for *bla*_CTX-M-14_, was determined to be resistant to AMP and CTX. The *tet* (B) or *tet* (A) was present in the seven *mcr-1* positive isolates, all displaying TET resistance. Additionally, all seven *mcr-1* positive isolates resistance to SXT, since these isolates carried trimethoprim resistance gene *dfrA12* or *dfrA14*, and sulfamethoxazole resistance gene *sul3* or *sul2* (except for 14EC001 carried *sul3* but no trimethoprim resistance gene). Six *mcr-1* positive isolates exhibited resistance to CM, but only five isolates were positive for *cmlA1*, *floR*, *floR2*, or *catB* encoding CM resistance. Isolate 14EC001 displaying the resistance to CM without *cml* or *floR* or *catB* determinants carried *oqxAB* gene, and a previous study showed that *oqxAB* could also confer the resistance to CM (Hansen et al., 2004). Six of seven *mcr-1* positive isolates present increased resistance to CIP. Among them, five isolates carried *qnrS*, *aac(6*′*)Ib-cr*, *oqxB*, *oqxA* determinants and one isolate (14EC020) had a chromosomal mutation in GyrA (Ser to Ala at amino acid position 83) that is known to cause ciprofloxacin resistance (Vila et al., 1994). Resistance to gentamicin was mediated mainly by the *aac (3*′*)* gene (Ramirez and Tolmasky, 2010), which is present in four of seven *mcr-1* positive isolates and displaying gentamicin resistance *aac(6*′*)Ib-cr* gene responsible for resistance to the aminoglycosides kanamycin, but susceptibility to gentamicin (Robicsek et al., 2006).

Furthermore, our analysis in the clinical isolates in China revealed that *mcr-1* is the only resistance gene found on these *mcr-1* carrying plasmids. These plasmids do not encode identified virulence factors. Recent studies demonstrated that increased expression of *mcr-1* in *E*. *coli* results in decreased bacterial growth, bacterial cell membrane impairment and attenuated virulence in animal infection model, suggesting that the expression of the *mcr-1* gene should be tightly regulated in *E. coli* (Yang et al., 2017). Furthermore, co-occurrence of *mcr-1* and other antimicrobial resistance genes on the same plasmid has been reported earlier (Bai et al., 2016; Haenni et al., 2016; Malhotra-Kumar et al., 2016; Yang et al., 2016; Zhi et al., 2016; Hadjadj et al., 2017; Zheng et al., 2017). However, these plasmids are usually larger than 200 kb and contain multiple plasmid replicons with more than one replication proteins (Supplementary Table S4). Previous studies have suggested that megaplasmids (>100 kb) are usually fused by smaller plasmids (Zheng et al., 2013). For example, plasmid pHNSHP45-2, which was isolated from porcine *E. coli* strain SHP45 where the first *mcr-1* gene was reported in 2015 is 251 kb (Zhi et al., 2016). Plasmid pHNSHP45 (64 kb), which was also present in *E. coli* strain SHP45 is the first reported *mcr-1* carrying plasmid and it contains only this resistance gene. Although *mcr-1* is the only resistance gene on *mcr-1* carrying plasmid in *E. coli* isolates obtained from 2013 to 2016 in this study, fusion of *mcr-1* carrying plasmid with other plasmids carrying multiple resistance genes in the future would promote the co-transfer of these antimicrobial resistance genes. Several studies have documented a link between the antimicrobial use and the development of antimicrobial resistance (Goossens et al., 2005; Bergman et al., 2009). Inappropriate use of one of these antimicrobial agents would potentially accelerate the co-transfer of several resistance genes. This emphasizes the need for ongoing monitoring of resistance patterns to ensure appropriate antibiotic use by clinicians.

## Author Contributions

HZ and BL conceptualized and designed the project. BL, XW, BK, XZ, WW, and YG did investigation, data curation, and data analysis. BL, XW, and HZ did supervision and visualization. BL, XW, and HZ wrote, reviewed, and edited the original draft.

## Conflict of Interest Statement

The authors declare that the research was conducted in the absence of any commercial or financial relationships that could be construed as a potential conflict of interest.

## Footnotes

1 http://soap.genomics.org.cn/

2 http://www.cbcb.umd.edu/software/glimmer/

3 http://www.genomicepidemiology.org/
