# Supplementary material for: Antimicrobial Resistance Profile of mcr-1 Positive Clinical Isolates of Escherichia coli in China From 2013 to 2016
Source: Front Microbiol. 2018 Oct 23;9:2514. doi: 10.3389/fmicb.2018.02514 (PMC6206212; doi:10.3389/fmicb.2018.02514)
Supplement: Supplementary file 1 [file Data_Sheet_1.docx]

**Supplementary File**

**Antimicrobial resistance profile of *mcr-1* positive clinical isolates of *Escherichia coli* in China from 2013-2016**

Baiyuan Li^1,2^, Bixia Ke^3^, Xuanyu Zhao^2,4^, Yunxue Guo^2^,

Weiquan Wang^2,4^, Xiaoxue Wang^2,4^, Honghui Zhu^1^*

^1^State Key Laboratory of Applied Microbiology Southern China, Guangdong Provincial Key Laboratory of Microbial Culture Collection and Application, Guangdong Microbial Culture Collection Center (GDMCC), Guangdong Open Laboratory of Applied Microbiology, Guangdong Institute of Microbiology, Guangzhou, China

^2^CAS Key Laboratory of Tropical Marine Bio-resources and Ecology, Guangdong Key Laboratory of Marine Materia Medica, RNAM Center for Marine Microbiology, South China Sea Institute of Oceanology, Chinese Academy of Science, Guangzhou 510301, China

^3^Center for Disease Control and Prevention of Guangdong Province, Guangzhou, China

^4^University of Chinese Academy of Sciences, Beijing 100049, China

***Corresponding author**: Honghui Zhu, Tel: (8620)-87137587, Fax: (8620) - 87137587, Email: [zhuhh@gdim.cn](mailto:zhuhh@gdim.cn).

**Keywords**: multidrug-resistant, clinical isolates, *Escherichia coli*, *mcr-1*, plasmid

**Table S1.** Primers used in this study.

| **Primers** | **Sequence** |
| --- | --- |
| CLR5-F(Liu et al., 2016) | CGGTCAGTCCGTTTGTTC |
| CLR5-R(Liu et al., 2016) | CTTGGTCGGTCTGTAGGG |
| 27F(Weisburg et al., 1991) | AGAGTTTGATCCTGGCTCA |
| 1492R(Weisburg et al., 1991) | GGTTACCTTGTTACGACTT |
| M13-F | CGCCAGGGTTTTCCCAGTCACGAC |
| RV-M | AGCGGATAACAATTTCACACAGGA |
| RhmT-F | TCACCTCGCAATGGTTTC |
| RhmT-R | AGTTGTGTCGGCATCGTC |
| pUC19-mcr-1F(chromosome) | CCGGAATTCGACACACCGGAGCAGGCACGTTTT |
| pUC19-mcr-1F(plasmid) | CCGGAATTCAATTTTGTTTCCACACAAAAACCA |
| pUC19-mcr-1R | CTTAGCGGCCGCATAGGTCA |

**Table S2.** Antimicrobial susceptibility profiles of 123 clinical *E*. *coli* isolates.

|  | **Antibiotics (µg/ml)** | | | | | | | | | | | |
| --- | --- | --- | --- | --- | --- | --- | --- | --- | --- | --- | --- | --- |
|  | **Polymyxins** | **Beta-lactam** | | | | | | **Fluoroquinolone** | **Aminoglycoside** | **Sulfamethoxazole -Trimethoprim** | **Phenicol** | **Tetracycline** |
| **Strains** | **Polymyxin B** | **Ampicillin** | **Cefoxitin** | **Ceftazidime** | **Imipenem** | **Cefotaxime** | **Cefepime** | **Ciprofloxacin** | **Gentamicin** | **SXT** | **Chloramphenicol** | **Tetracycline** |
| 13EC001 | 1 | >64 | 4 | <0.25 | 0.25 | <0.25 | <0.5 | <0.125 | <0.5 | <0.13/2.4 | >64 | 64 |
| 13EC002 | 1 | <2 | 2 | <0.25 | <0.125 | <0.25 | <0.5 | <0.125 | 1 | <0.13/2.4 | 4 | 1 |
| 13EC003 | 1 | <2 | 4 | <0.25 | <0.125 | <0.25 | <0.5 | <0.125 | <0.5 | <0.13/2.4 | 8 | >64 |
| 13EC004 | 0.5 | <2 | 8 | <0.25 | <0.125 | <0.25 | <0.5 | 0.25 | <0.5 | <0.13/2.4 | 8 | 2 |
| 13EC005 | 0.5 | 4 | 8 | 0.5 | <0.125 | <0.25 | <0.5 | 0.25 | <0.5 | <0.13/2.4 | 8 | 2 |
| 13EC006 | 0.5 | >64 | 4 | 16 | 0.25 | >32 | >64 | 0.25 | >64 | >16/304 | 8 | 2 |
| 13EC007 | 0.5 | >64 | 4 | <0.25 | <0.125 | <0.25 | <0.5 | 2 | 32 | >16/304 | 32 | 32 |
| 13EC008 | 0.5 | <2 | 4 | <0.25 | <0.125 | <0.25 | <0.5 | 0.5 | <0.5 | >16/304 | 64 | 32 |
| 13EC009 | 1 | >64 | 2 | <0.25 | <0.125 | <0.25 | <0.5 | 0.25 | 1 | >16/304 | 4 | 1 |
| 13EC010 | 0.5 | 4 | 4 | <0.25 | <0.125 | <0.25 | <0.5 | <0.125 | <0.5 | <0.13/2.4 | 8 | 2 |
| 13EC011 | 1 | >64 | <0.25 | <0.25 | 0.5 | <0.25 | <0.5 | <0.125 | 64 | >16/304 | 8 | 64 |
| 13EC012 | 1 | <2 | <0.25 | <0.25 | <0.125 | <0.25 | <0.5 | 0.25 | <0.5 | 1.0/19 | 8 | 64 |
| 13EC013 | 1 | <2 | <0.25 | <0.25 | <0.125 | <0.25 | <0.5 | 0.25 | <0.5 | 0.5/9.5 | 8 | >64 |
| 13EC014 | <0.25 | >64 | <0.25 | <0.25 | <0.125 | <0.25 | <0.5 | 4 | <0.5 | 0.25/4.75 | 64 | >64 |
| 13EC015 | <0.25 | >64 | <0.25 | <0.25 | <0.125 | <0.25 | <0.5 | 4 | 16 | >16/304 | 64 | 64 |
| 14EC001 | 8 | <2 | 4 | <0.25 | <0.125 | <0.25 | <0.5 | <0.125 | <0.5 | 1.0/19 | 16 | >64 |
| 14EC002 | <0.25 | >64 | 8 | <0.25 | <0.125 | <0.25 | <0.5 | <0.125 | <0.5 | >16/304 | >64 | 64 |
| 14EC003 | <0.25 | >64 | 4 | <0.25 | <0.125 | <0.25 | <0.5 | <0.125 | <0.5 | >16/304 | 64 | 32 |
| 14EC004 | <0.25 | >64 | 4 | <0.25 | <0.125 | <0.25 | <0.5 | 0.25 | 64 | >16/304 | >64 | 64 |
| 14EC005 | 0.5 | <2 | 2 | <0.25 | <0.125 | <0.25 | <0.5 | <0.125 | <0.5 | <0.13/2.4 | 2 | 1 |
| 14EC006 | 0.5 | <2 | 4 | <0.25 | <0.125 | <0.25 | <0.5 | 0.25 | <0.5 | <0.13/2.4 | 4 | 2 |
| 14EC007 | 4 | >64 | 4 | <0.25 | <0.125 | <0.25 | <0.5 | 0.25 | <0.5 | >16/304 | 4 | 32 |
| 14EC008 | <0.25 | >64 | 4 | <0.25 | <0.125 | <0.25 | <0.5 | 0.25 | <0.5 | <0.13/2.4 | 4 | >64 |
| 14EC009 | <0.25 | >64 | 4 | <0.25 | <0.125 | <0.25 | <0.5 | 0.5 | <0.5 | 0.5/9.5 | 4 | 64 |
| 14EC010 | 0.5 | <2 | 4 | <0.25 | <0.125 | <0.25 | <0.5 | <0.125 | 16 | >16/304 | >64 | 32 |
| 14EC011 | <0.25 | >64 | 8 | 2 | <0.125 | 32 | 8 | <0.125 | 32 | 16/304 | 4 | 4 |
| 14EC012 | 0.5 | >64 | 8 | <0.25 | <0.125 | <0.25 | <0.5 | 0.25 | <0.5 | >16/304 | 8 | 64 |
| 14EC013 | 1 | >64 | 4 | <0.25 | 0.25 | <0.25 | <0.5 | 0.5 | <0.5 | 0.25/4.75 | 4 | 64 |
| 14EC014 | 0.5 | >64 | 8 | 2 | 0.25 | >32 | 32 | 0.25 | 64 | >16/304 | >64 | 64 |
| 14EC015 | 0.5 | >64 | 4 | <0.25 | <0.125 | <0.25 | <0.5 | 0.25 | <0.5 | >16/304 | 4 | 1 |
| 14EC016 | 0.5 | >64 | 4 | 1 | <0.125 | >32 | 8 | 2 | >64 | >16/304 | 4 | 64 |
| 14EC017 | 4 | >64 | 4 | 1 | 0.25 | 0.5 | <0.5 | 8 | >64 | >16/304 | >64 | 64 |
| 14EC018 | 1 | >64 | 2 | <0.25 | <0.125 | <0.25 | <0.5 | 0.25 | 1 | >16/304 | 4 | 64 |
| 14EC019 | 0.5 | >64 | 2 | 0.5 | <0.125 | 32 | 4 | 0.25 | <0.5 | <0.13/2.4 | 8 | 64 |
| 14EC020 | 4 | >64 | 4 | <0.25 | <0.125 | <0.25 | <0.5 | 1 | 32 | >16/304 | >64 | 64 |
| 14EC021 | 0.5 | <2 | 4 | <0.25 | <0.125 | <0.25 | <0.5 | <0.125 | <0.5 | <0.13/2.4 | 4 | 1 |
| 14EC022 | 0.5 | >64 | >32 | 1 | <0.125 | 8 | 2 | <0.125 | 32 | 0.25/4.75 | 8 | 2 |
| 14EC023 | 0.5 | <2 | 2 | <0.25 | <0.125 | <0.25 | <0.5 | <0.125 | <0.5 | <0.13/2.4 | 4 | 2 |
| 14EC024 | 0.5 | >64 | 8 | 0.25 | <0.125 | <0.25 | <0.5 | 0.25 | <0.5 | >16/304 | >64 | 64 |
| 14EC025 | 0.5 | >64 | 4 | 16 | <0.125 | >32 | 16 | >16 | 64 | >16/304 | 8 | 64 |
| 14EC026 | 1 | >64 | 8 | <0.25 | <0.125 | <0.25 | <0.5 | 0.25 | 32 | <0.13/2.4 | 4 | 64 |
| 14EC027 | 1 | <2 | 4 | <0.25 | <0.125 | <0.25 | <0.5 | 0.25 | <0.5 | >16/304 | 4 | 64 |
| 14EC028 | 0.5 | <2 | 8 | <0.25 | <0.125 | <0.25 | <0.5 | <0.125 | <0.5 | <0.13/2.4 | 8 | 2 |
| 14EC029 | 4 | >64 | 4 | 1 | 0.25 | >32 | 2 | 1 | 8 | >16/304 | >64 | 64 |
| 14EC030 | <0.25 | >64 | 4 | 0.5 | <0.125 | >32 | 4 | 0.25 | <0.5 | <0.13/2.4 | 4 | 1 |
| 14EC031 | 0.5 | >64 | 8 | <0.25 | <0.125 | <0.25 | <0.5 | 0.5 | 64 | <0.13/2.4 | 8 | 64 |
| 14EC032 | 0.5 | >64 | 8 | 16 | 0.25 | >32 | 64 | >16 | >64 | >16/304 | >64 | 64 |
| 14ECO33 | 4 | >64 | 2 | <0.25 | 0.25 | <0.25 | <0.5 | 1 | <0.5 | >16/304 | 16 | 64 |
| 14EC034 | 0.5 | <2 | 4 | <0.25 | <0.125 | <0.25 | <0.5 | <0.125 | 1 | <0.13/2.4 | 8 | 2 |
| 14EC035 | 4 | <2 | 2 | <0.25 | <0.125 | <0.25 | <0.5 | 0.25 | 16 | >16/304 | 4 | >64 |
| 14EC036 | 0.5 | >64 | 8 | <0.25 | <0.125 | <0.25 | <0.5 | 0.25 | <0.5 | <0.13/2.4 | >64 | 64 |
| 14EC037 | 0.5 | <2 | 4 | <0.25 | <0.125 | <0.25 | <0.5 | <0.125 | <0.5 | <0.13/2.4 | 8 | 2 |
| 14EC038 | <0.25 | <2 | 2 | <0.25 | <0.125 | <0.25 | <0.5 | 0.25 | <0.5 | >16/304 | 4 | 32 |
| 14EC039 | 0.5 | >64 | 2 | <0.25 | <0.125 | <0.25 | <0.5 | 0.25 | <0.5 | >16/304 | 4 | 64 |
| 14EC040 | 0.5 | >64 | 4 | <0.25 | 0.25 | <0.25 | <0.5 | 1 | <0.5 | 0.5/9.5 | 4 | 64 |
| 14EC041 | 1 | >64 | >32 | >32 | <0.125 | >32 | >64 | >16 | 32 | >16/304 | >64 | >64 |
| 14EC042 | 0.5 | >64 | >32 | >32 | 0.25 | >32 | >64 | >16 | 32 | >16/304 | >64 | >64 |
| 14EC043 | 4 | 64 | 32 | 8 | <0.125 | 4 | <0.5 | 4 | 8 | >16/304 | 64 | >64 |
| 14EC044 | 0.5 | >64 | 4 | <0.25 | 0.25 | <0.25 | <0.5 | <0.125 | >64 | >16/304 | 4 | 64 |
| 14EC045 | >32 | 64 | >32 | >32 | >16 | >32 | >64 | >16 | >64 | >16/304 | 8 | >64 |
| 14EC046 | 0.5 | >64 | 32 | >32 | <0.125 | >32 | >64 | 16 | <0.5 | 16/304 | >64 | >64 |
| 14EC047 | 0.5 | >64 | 2 | <0.25 | <0.125 | <0.25 | <0.5 | 0.5 | <0.5 | >16/304 | >64 | 32 |
| 14EC048 | 0.5 | 64 | 32 | 4 | <0.125 | 8 | <0.5 | 0.25 | 64 | >16/304 | 8 | 1 |
| 14EC049 | 0.5 | <2 | 2 | <0.25 | <0.125 | <0.25 | <0.5 | 0.25 | 1 | 1/19 | 4 | 64 |
| 14EC050 | 0.5 | >64 | 8 | 1 | <0.125 | >32 | 4 | 1 | 16 | >16/304 | >64 | 64 |
| 14EC051 | 0.5 | >64 | 4 | <0.25 | 0.25 | <0.25 | <0.5 | <0.125 | >64 | >16/304 | 16 | 64 |
| 14EC052 | 0.5 | >64 | 8 | 0.5 | <0.125 | <0.25 | <0.5 | <0.125 | <0.5 | <0.13/2.4 | 8 | 64 |
| 14EC053 | 0.5 | >64 | 8 | <0.25 | <0.125 | <0.25 | <0.5 | 1 | 64 | >16/304 | 8 | >64 |
| 14EC054 | 0.5 | >64 | 8 | 16 | <0.125 | >32 | 32 | >16 | <0.5 | >16/304 | >64 | 64 |
| 14EC055 | 0.5 | >64 | >32 | >32 | <0.125 | >32 | 64 | <0.125 | <0.5 | >16/304 | 8 | >64 |
| 14EC056 | 0.5 | >64 | 8 | 4 | <0.125 | >32 | 64 | <0.125 | 1 | >16/304 | 4 | 32 |
| 14EC057 | 0.5 | 32 | 8 | <0.25 | <0.125 | <0.25 | <0.5 | 1 | <0.5 | <0.13/2.4 | >64 | 64 |
| 15EC001 | <0.25 | <2 | 4 | <0.25 | 0.125 | <0.25 | <0.5 | <0.125 | <0.5 | <0.13/2.4 | 8 | 2 |
| 15EC002 | 0.5 | >64 | 4 | <0.25 | <0.125 | <0.25 | <0.5 | <0.125 | 1 | <0.13/2.4 | 8 | 64 |
| 15EC003 | <0.25 | <2 | 2 | <0.25 | <0.125 | <0.25 | <0.5 | <0.125 | <0.5 | 0.25/4.75 | 4 | 64 |
| 15EC004 | 0.5 | >64 | 4 | <0.25 | <0.125 | <0.25 | <0.5 | 0.5 | <0.5 | >16/304 | 8 | >64 |
| 15EC005 | 0.5 | >64 | 4 | <0.25 | <0.125 | <0.25 | <0.5 | 0.25 | 1 | >16/304 | 4 | 64 |
| 15EC006 | 0.5 | >64 | 4 | <0.25 | <0.125 | <0.25 | <0.5 | 0.25 | <0.5 | >16/304 | 4 | 1 |
| 15EC007 | 0.5 | >64 | 4 | <0.25 | <0.125 | <0.25 | <0.5 | 0.5 | <0.5 | 0.25/4.75 | >64 | 8 |
| 15EC008 | 0.5 | >64 | <0.25 | <0.25 | <0.125 | <0.25 | <0.5 | 0.25 | <0.5 | 0.25/4.75 | >64 | 64 |
| 15EC009 | 0.5 | >64 | 8 | <0.25 | 0.25 | <0.25 | <0.5 | 0.25 | 64 | 0.25/4.75 | 8 | 64 |
| 15EC010 | 1 | <2 | 4 | <0.25 | <0.125 | <0.25 | <0.5 | 1 | <0.5 | >16/304 | >64 | 64 |
| 15EC011 | 0.5 | 0.5 | 4 | <0.25 | <0.125 | <0.25 | <0.5 | <0.125 | <0.5 | <0.13/2.4 | 8 | 1 |
| 15EC012 | 0.5 | <2 | 4 | <0.25 | <0.125 | <0.25 | <0.5 | <0.125 | <0.5 | <0.13/2.4 | 8 | 1 |
| 15EC013 | 0.5 | >64 | 4 | <0.25 | <0.125 | <0.25 | <0.5 | <0.125 | <0.5 | <0.13/2.4 | 4 | 64 |
| 15EC014 | 0.5 | <2 | 8 | <0.25 | <0.125 | <0.25 | <0.5 | 0.25 | <0.5 | <0.13/2.4 | 8 | 2 |
| 15EC015 | 0.5 | >64 | 2 | <0.25 | 0.25 | 8 | 16 | <0.125 | 64 | >16/304 | 8 | 1 |
| 15EC016 | 0.5 | <2 | 8 | <0.25 | <0.125 | <0.25 | <0.5 | <0.125 | 1 | <0.13/2.4 | 8 | 2 |
| 15EC017 | 0.5 | >64 | 4 | <0.25 | <0.125 | <0.25 | <0.5 | 0.25 | <0.5 | 0.5/4.75 | 8 | 64 |
| 15EC018 | 0.5 | 0.5 | 4 | <0.25 | <0.125 | <0.25 | <0.5 | 0.5 | <0.5 | >16/304 | >64 | 64 |
| 15EC019 | 0.5 | >64 | 4 | <0.25 | <0.125 | <0.25 | <0.5 | <0.125 | 64 | >16/304 | 4 | 32 |
| 15EC020 | <0.25 | <2 | <0.25 | <0.25 | <0.125 | <0.25 | <0.5 | <0.125 | <0.5 | >16/304 | 8 | 2 |
| 15EC021 | 0.5 | >64 | 8 | 8 | <0.125 | >32 | 64 | <0.125 | <0.5 | <0.13/2.4 | 8 | 2 |
| 15EC022 | 0.5 | <2 | 4 | <0.25 | <0.125 | <0.25 | <0.5 | <0.125 | <0.5 | <0.13/2.4 | 4 | 1 |
| 15EC023 | 0.5 | >64 | 4 | 2 | <0.125 | >32 | 16 | 0.5 | 64 | >16/304 | 4 | 64 |
| 15EC024 | 0.5 | >64 | 4 | <0.25 | <0.125 | <0.25 | <0.5 | 0.25 | <0.5 | >16/304 | 8 | 64 |
| 15EC025 | 1 | >64 | 4 | <0.25 | <0.125 | <0.25 | <0.5 | 0.5 | 64 | <0.13/2.4 | 4 | 64 |
| 15EC026 | 1 | >64 | 4 | 8 | <0.125 | >32 | 64 | <0.125 | 1 | <0.13/2.4 | 8 | 2 |
| 15EC027 | 1 | <2 | 2 | <0.25 | <0.125 | <0.25 | <0.5 | <0.125 | <0.5 | <0.13/2.4 | 2 | 1 |
| 15EC028 | 1 | >64 | 4 | <0.25 | <0.125 | <0.25 | <0.5 | 0.25 | <0.5 | >16/304 | >64 | 64 |
| 15EC029 | 1 | <2 | 2 | <0.25 | <0.125 | <0.25 | <0.5 | <0.125 | <0.5 | <0.13/2.4 | 4 | 1 |
| 15EC030 | 0.5 | >64 | 4 | 4 | <0.125 | 32 | 4 | <0.125 | <0.5 | <0.13/2.4 | 4 | 1 |
| 15EC031 | 0.5 | >64 | 16 | 16 | <0.125 | >32 | >64 | >16 | 32 | >16/304 | >64 | >64 |
| 15EC032 | 1 | <2 | 4 | <0.25 | <0.125 | <0.25 | <0.5 | <0.125 | <0.5 | <0.13/2.4 | 2 | 1 |
| 15EC033 | 1 | >64 | 4 | <0.25 | <0.125 | <0.25 | <0.5 | <0.125 | 64 | >16/304 | 4 | 2 |
| 15EC034 | 0.5 | >64 | 4 | <0.25 | <0.125 | >32 | >64 | 0.25 | 32 | >16/304 | >64 | >64 |
| 15EC035 | 0.5 | >64 | 4 | 4 | <0.125 | 32 | 4 | 0.25 | 16 | >16/304 | >64 | 64 |
| 15EC036 | 1 | >64 | 4 | 0.5 | 0.25 | >32 | 1 | <0.125 | 32 | 0.5/9.5 | 8 | 2 |
| 15EC037 | 0.5 | >64 | 8 | 4 | <0.125 | >32 | 4 | 0.25 | 8 | >16/304 | >64 | 64 |
| 16EC001 | 0.5 | <2 | <0.25 | <0.25 | <0.125 | <0.25 | <0.5 | 0.25 | <0.5 | <0.13/2.4 | 4 | <0.5 |
| 16EC002 | 0.5 | <2 | <0.25 | <0.25 | <0.125 | <0.25 | <0.5 | <0.125 | <0.5 | <0.13/2.4 | 4 | 1 |
| 16EC003 | 1 | <2 | <0.25 | <0.25 | <0.125 | <0.25 | <0.5 | 16 | <0.5 | >16/304 | 4 | 32 |
| 16EC004 | 0.5 | >64 | <0.25 | <0.25 | <0.125 | <0.25 | <0.5 | 0.25 | 64 | <0.13/2.4 | 8 | 2 |
| 16EC005 | <0.25 | >64 | <0.25 | <0.25 | <0.125 | <0.25 | <0.5 | <0.125 | 64 | <0.13/2.4 | 4 | 32 |
| 16EC006 | <0.25 | <2 | 2 | <0.25 | <0.125 | <0.25 | <0.5 | <0.125 | <0.5 | <0.13/2.4 | 4 | 1 |
| 16EC007 | 0.5 | <2 | 4 | <0.25 | <0.125 | 2 | <0.5 | 0.25 | 2 | <0.13/2.4 | 4 | 2 |
| 16EC008 | 0.5 | >64 | 8 | 1 | 1 | >32 | 4 | <0.125 | 1 | >16/304 | 4 | 64 |
| 16EC009 | 0.5 | <2 | 4 | <0.25 | <0.125 | <0.25 | <0.5 | <0.125 | <0.5 | <0.13/2.4 | 4 | 2 |
| 16EC010 | <0.25 | >64 | 4 | <0.25 | <0.125 | <0.25 | <0.5 | 0.25 | 64 | <0.13/2.4 | 4 | 64 |
| 16EC011 | 0.5 | >64 | 2 | <0.25 | <0.125 | <0.25 | <0.5 | <0.125 | <0.5 | >16/304 | 4 | 1 |
| 16EC012 | 0.5 | >64 | 4 | 0.5 | <0.125 | 32 | 16 | 0.25 | 1 | 0.5/9.5 | 4 | 1 |
| 16EC013 | 0.5 | >64 | 4 | 0.5 | <0.125 | 4 | 1 | 0.25 | <0.5 | >16/304 | 4 | 32 |
| 16EC014 | 0.5 | >64 | 8 | 2 | <0.125 | >32 | 32 | 0.25 | 1 | >16/304 | >64 | 64 |

**Table S3.** Distribution of antimicrobial resistance genes among seven *mcr-1* positive *E*. *coli* isolates.

|  |  | **Beta-lactam** | **Colistin** | **Fluoroquinolone** | **Aminoglycoside** | **Sulphonamide** | **Trimethoprim** | **Phenicol** | **Tetracycline** | **Fosfomycin** | **MLS^a^** | **Rifampicin** |
| --- | --- | --- | --- | --- | --- | --- | --- | --- | --- | --- | --- | --- |
| **Strains** | **Plasmid/**  **Chromosme** | **AMP, FOX, CAZ, IPM, CTX, FEP** | **colistin** | **CIP** | **GEN** | **SXT** | **SXT** | **CM** | **TET** | **Rosfomycin** | **MLS** | **Rifampicin** |
| **14EC001** | **p14EC001a** | ***-*^b^** | *mcr-1* | - | - | - | - | - | - | - | ***-*** | ***-*** |
|  | **p14EC001b** | ***-*** | *-* | *-* | *-* | *-* | - | - | *tet*(B) | - | ***-*** | ***-*** |
|  | **p14EC001c** | ***-*** | *-* | *oqxB*, *oqxA* | *aph(3')-Ia* | *sul3* | - | - | *-* | - | *mef* (B) | ***-*** |
|  | **Chromosome** | ***-*** | *mcr-1* | *-* | *-* | *-* | - | - | *-* | - | *-* | ***-*** |
| **14EC007** | **p14EC007a** | ***-*** | *mcr-1* | - | - | - | - | - | - | - | *-* | ***-*** |
|  | **p14EC007b** | *bla*_TEM-1B_ | *-* | *qnrS1* | *aadA* | *sul3* | *dfrA12* | - | *tet*(A) | - | ***-*** | ***-*** |
|  | **Chromosome** | *-* | *-* | *-* | *-* | *-* | *-* | *-* | *-* | - | ***-*** | *-* |
| **14EC017** | **p14EC017a** | ***-*** | *mcr-1* | *-* | *-* | *-* | *-* | *-* | *-* | *-* | *-* | *-* |
|  | **p14EC017b** | *bla*_TEM-1B_ | *-* | *oqxA*, *oqxB* | *aac(3)-IId*, *aadA1*, *aadA, aph(3')-IIa* | *sul3* | *dfrA12* | *cmlA1* | *tet*(A) | - | ***-*** | ***-*** |
|  | **p14EC017c** | *-* | *-* | *-* | *-* | *-* | *-* | *-* | *-* | - | ***-*** | ***-*** |
|  | **Chromosome** | ***-*** | *-* | - | *strB*, *strA*, | *sul2* | *-* | *floR* | *tet*(A) | *-* | *-* | *-* |
| **14EC020** | **p14EC020a** | ***-*** | *mcr-1* | - | *-* | *-* | *-* | *-* | *-* | - | ***-*** | ***-*** |
|  | **p14EC020b** | *bla*_TEM-1A_ | *-* | - | *aph(3')-Ia*, *strB*, *strA*, *aac(3)-IId* | *sul3*, *sul2* | *dfrA14* | *floR2* | *tet*(A) | *-* | *-* | ***-*** |
|  | **Chromosome** | *-* | *-* | *-* | *-* | *-* | *-* | *-* | *-* | - | ***-*** | *-* |
| **14EC029** | **p14EC029a** | ***-*** | *mcr-1* | *-* | *-* | *-* | *-* | *-* | *-* | *-* | *-* | *-* |
|  | **p14EC029b** | *bla*_CTX-M-14_ | *-* | *oqxA*, *oqxB* | *aac(3)-Iva*, *aph(4)-Ia*, *aadA2*, *aadA1, aph(3')-Ia* | *sul1-3* | *dfrA12* | *floR,cmlA1* | *-* | *fosA* | *mph*(A) | ***-*** |
|  | **p14EC029c** | *-* | *-* | *qnrS1* | *aadA2*, *aadA1, aph(3')-Ia* | *sul3* | *dfrA12* | *cmlA1* | *tet*(A) | *-* | *mef*(B) | ***-*** |
|  | **p14EC029d** | *-* | *-* | *-* | *-* | *-* | *-* | *-* | *-* | - | ***-*** | *-* |
|  | **p14EC029e** | *-* | *-* | *-* | *-* | *-* | *-* | *-* | *-* | - | ***-*** | *-* |
|  | **Chromosome** | *-* | *-* | *-* | *-* | *-* | *-* | *-* | *-* | - | ***-*** | *-* |
| **14EC033** | **p14EC033a** | - | *mcr-1* | *-* | *-* | *-* | *-* | - | - | *-* | *-* | ***-*** |
|  | **p14EC033b** | *-* | *mcr-1* | *-* | *-* | *-* | *-* | *-* | *-* | - | ***-*** | ***-*** |
|  | **p14EC033c** | *-* | *-* | *-* | *-* | *-* | *-* | *-* | *-* | - | ***-*** | *-* |
|  | **p14EC033d** | *-* | *-* | *-* | *-* | *-* | *-* | *-* | *-* | - | ***-*** | *-* |
|  | **p14EC033e** | *-* | *-* | *-* | *-* | *-* | *-* | *-* | *-* | - | ***-*** | *-* |
|  | **p14EC033f** | *bla*_OXA-1_ | *-* | *qnrS2*, *aac(6')Ib-cr*, *oqxB*, *oqxA* | *aac(6')Ib-cr*, *aadA2*, *aph(3')-IIa*, | *sul3* | *dfrA12* | *catB* | *tet*(A) | *-* | *-* | *arr-3* |
|  | **p14EC033g** | *-* | *-* | *-* | *-* | *-* | *-* | *-* | *-* | *-* | ***-*** | *-* |
|  | **Chromosome** | *-* | *-* | *-* | *-* | *-* | *-* | *-* | *-* | *-* | ***-*** | *-* |
| **14EC047** | **p14EC047a** | ***-*** | *mcr-1* | - | *-* | *-* | *-* | *-* | *-* | *-* | *-* | *-* |
|  | **p14EC047b** | *-* | *-* | *-* | *aadA1*, *aadA2*, | *sul3* | *dfrA12* | *cmlA1*, f*loR* | *tet*(A) | *-* | ***-*** | ***-*** |
|  | **p14EC047c** | *-* | *-* | *-* | *-* | *-* | *-* | *-* | *-* | *-* | ***-*** | *-* |
|  | **Chromosome** | *-* | *-* | *-* | *-* | *-* | *-* | *-* | *-* | *-* | ***-*** | *-* |

^a^, Macrolide, Lincosamide and Streptogramin B; -^b^, undetected.

**Table S4.** Characteristics of *mcr-1* carrying plasmids.

| **Plasmids** | **Accession**  **Number** | **Origin** | **Size (bp)** | **Inc types** | **Replication Proteins** | **Resistance Genes** | **Reference** |
| --- | --- | --- | --- | --- | --- | --- | --- |
| pLH30-mcr1 | NZ_CM008265 | human | 223,898 | IncHI2/IncHI2A | RepB/RepB1 | *mcr-1*, *bla*_TEM-217_, *cmlA1*, *floR*, *aph(3')-Ia*, *aadA2*, *sulIII*, *dfrA17,* *mefB* | (Hadjadj et al., 2017) |
| pLH57-mcr1 | NZ_CM008264 | human | 218,800 | IncFII/IncFIB/IncFIC (FII)/IncN/IncHI2A | RepA2/RepA, RepB/RepB1/ RepA/RepA1/Rep, | *mcr-1*, *bla*_TEM-217_, *tet*(A), *tet*(R), *strA*, *strB*, *aph**(3')-Ib*, a*ph (6')-Id*, *sulII*, d*frA14* | (Hadjadj et al., 2017) |
| pLH1-mcr1 | NZ_CM008267 | human | 248,201 | IncFIB (K)/IncHI2/ IncHI2A | RepB/RepB1/Rep | *mcr-1*, *bla*_TEM-217_, *tet*(A), *tet*(R), *oqxA*, *aadA2*,  *oqxB*, *sulI*, *sulIII*, *dfrA12* | (Hadjadj et al., 2017) |
| p1RC4-mcr1 | NZ_CM008266 | human | 239,098 | IncHI2A/IncHI2 | RepB/RepB1 | *mcr-1*, *bla*_TEM-217_, *tet*(A), *tet*(R), *floR*, *aph(3')-Ia*, *aph(3')-Ib*, *aadA1*, *strA*, *sulIII*, *dfrA14* | (Hadjadj et al., 2017) |
| pHNSHP45-2 | KU341381 | animal | 251,493 | IncHI2A/IncHI2 | RepHI2/RepA | *aph(4)-Ia*, *aac(3)-IVa*, *aadA2*, *aph(3')-Ia*, *aadA1*, *aadA2*, *bla*_CTX-M-14_, *mcr-1*, *oqxB*, *oqxA*, *fosA3*, *floR*, *cmlA1*, *sul2*, *sul1*, *sul3* , *dfrA12* | (Zhi et al., 2016) |
| pEC1002-MCR | CP021205 | human | 63,392 | IncI2 | Rep | *mcr-1* | (Zheng et al., 2017) |
| pHNZ319S^a^ | KX257482 | animal | 54,494 | -^b^ | - | *mcr-1* |  |
| pICBEC72Hmcr | CP015977 | human | 33,304 | IncX4 | Rep | *mcr-1* | (Fernandes et al., 2016) |
| pmcr1_IncX4 | KU761327 | human | 33,287 | IncX4 | - | *mcr-1* | (Li et al., 2016) |
| pHNSHP45 | KP347127 | animal | 64,015 | IncI2 | RepA | *mcr-1* | (Liu et al., 2016) |

^a^ Direct Submission (unpublished); -^b^ undetected.

**Figure S1.** Screening the *mcr-1* positive isolates by PCR with primer pair CLR5-F/CLR5-R. M, DNA marker DS 5000; PC, *E*. *coli* C600/pHNSHP45 as positive control; a-g, *mcr-1* positive isolates.


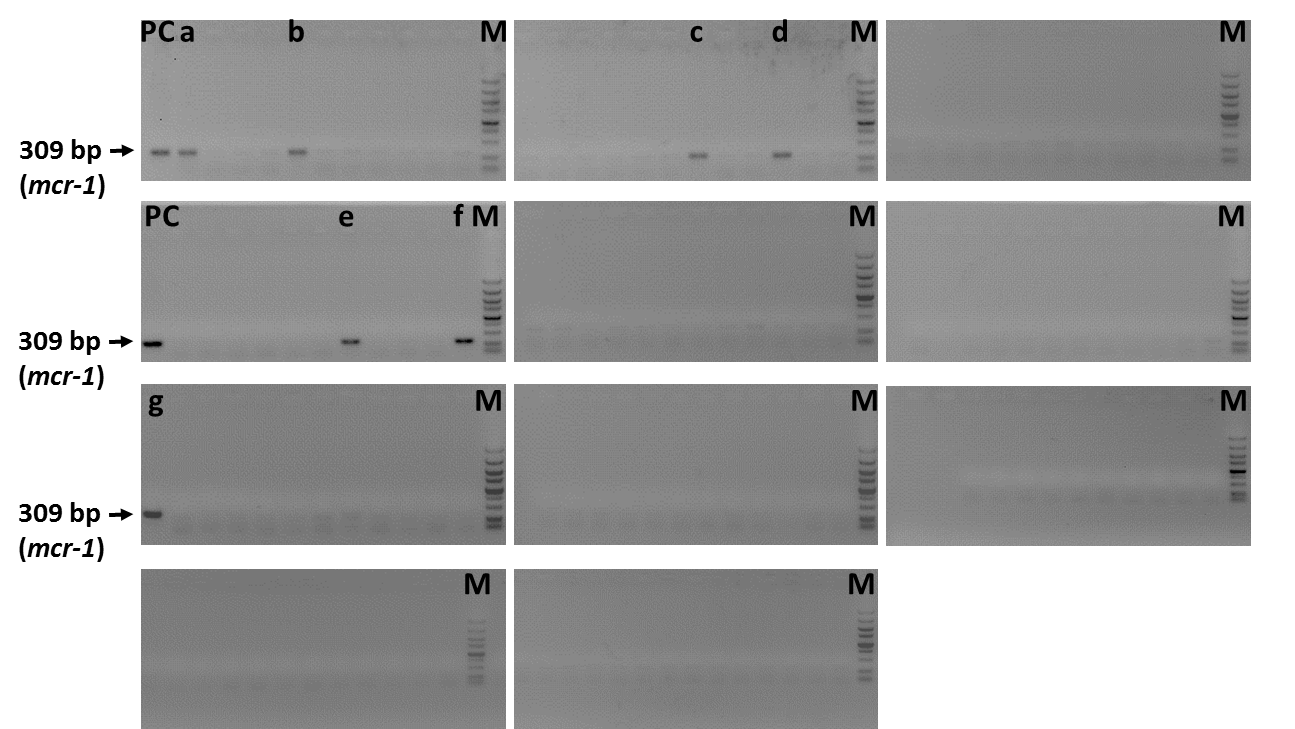


**Figure S2.** Schematic representation of plasmid-encoded and chromosome-encoded *mcr-1* -*pap2* region in the 14EC001 and comparison of the inserted region to *E*. *coli* MG1655. (A) Comparative analysis of genetic structures of *mcr-1* and flanking region in plasmids and the chromosomes of 14EC001, 14EC007, *E. coli* K-12 MG1655. In the 14EC001 host chromosome, the *mcr-1*-*pap2* region is inserted in the coding region of the *rhmT* gene. The *rhmRDTA* operon in *E. coli* K-12 MG1655 is shown. The interrupted *rhmT* gene in 14EC001 is marked with *rhmT’* and *rhmT’’*. Regions of homology between sequences (> 84%) are indicated by the graded shading. (B) The presence of an extra 2.6 kb fragment inside the *rhmT* gene in strain 14EC001 was confirmed by PCR using primers flanking the *rhmT* gene (lane 3). The other 4 strains (lanes 2, 4, 5 and 6) were used as negative controls. Lane 1 indicates the DNA marker. (C) The absence of the IS*Apl1* from the *mcr-1*-containing cassette was detected in the host chromosome of 14EC001. The two DNA fragments, one from p14EC001a and one from the host chromosome of 14EC001 that were cloned into pUC19 vector are bordered with vertical dotted lines.


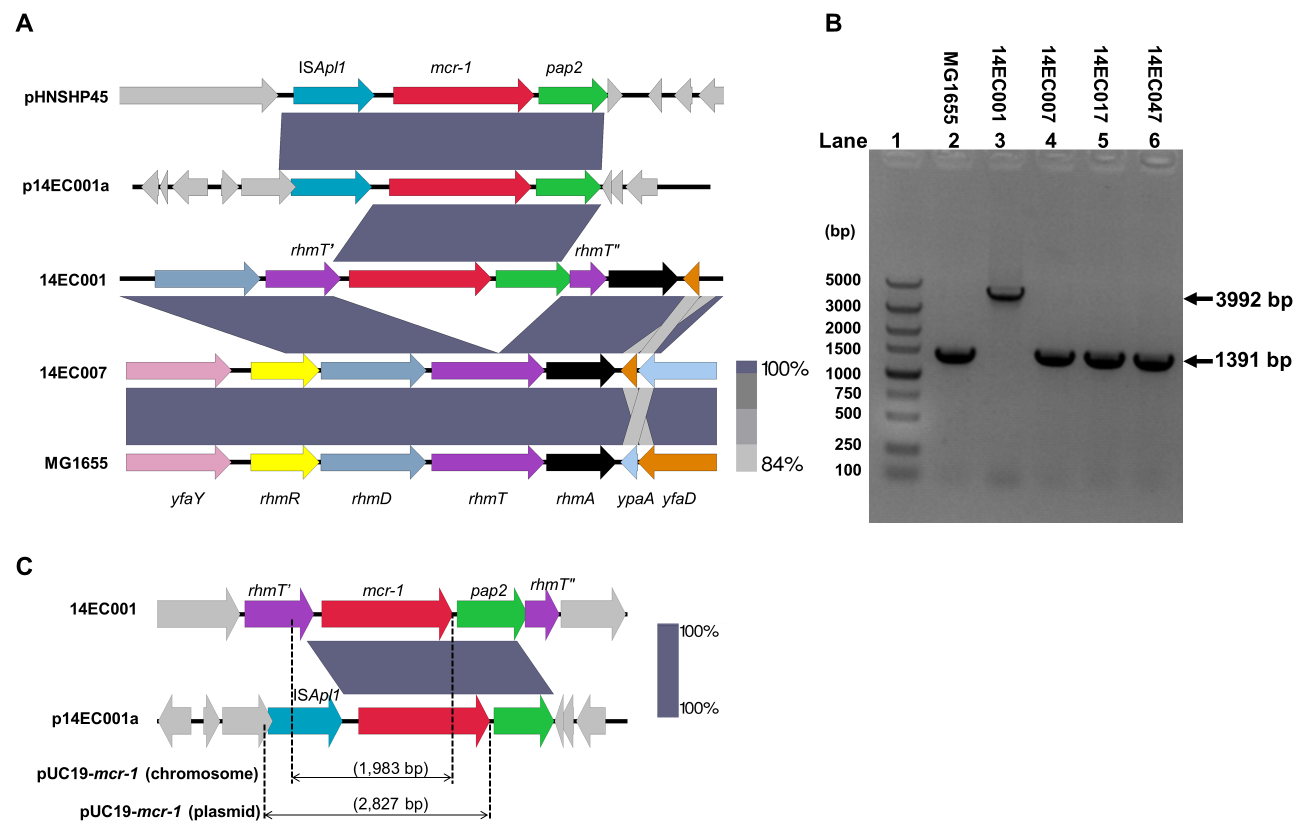


**Figure S3.** Linear characterization of plasmid pHNSHP45-2 carrying multidrug resistance genes and pHNZ319S carrying *mcr-1*. Antimicrobial resistance genes were presented and each class of antibiotic was marked with different colors. Grey regions between plasmids indicate nucleotide identity (67-100%) by BLASTN. Arrows indicate predicted ORFs.


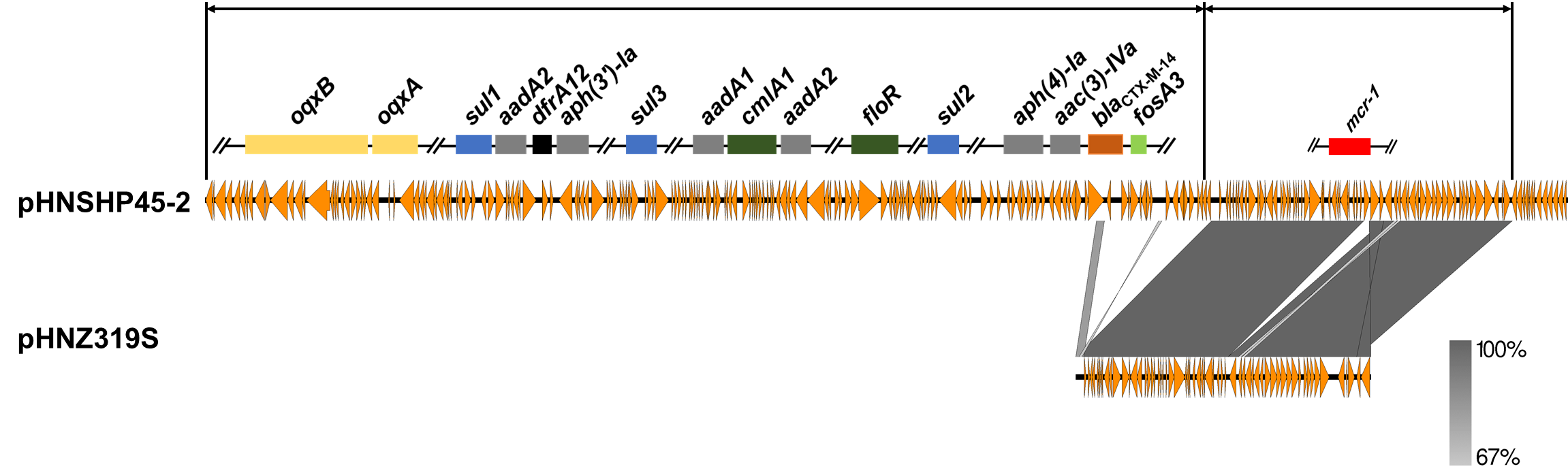


Fernandes, M.R., McCulloch, J.A., Vianello, M.A., Moura, Q., Perez-Chaparro, P.J., Esposito, F., et al. (2016). First report of the globally disseminated IncX4 plasmid carrying the *mcr-1* gene in a colistin-resistant *Escherichia coli* sequence type 101 isolate from a human infection in Brazil. *Antimicrob. Agents Chemother.* 60(10)**,** 6415-6417. doi: 10.1128/Aac.01325-16.

Hadjadj, L., Riziki, T., Zhu, Y., Li, J., Diene, S.M., and Rolain, J.M. (2017). Study of *mcr-1* gene-mediated colistin resistance in Enterobacteriaceae isolated from humans and animals in different countries. *Genes* 8(12), 394.. doi: ARTN 39410.3390/genes8120394.

Li, A.Q., Yang, Y., Miao, M.H., Chavda, K.D., Mediavilla, J.R., Xie, X.F., et al. (2016). Complete sequences of *mcr-1*-harboring plasmids from extended-spectrum-beta-lactamase- and carbapenemase-producing Enterobacteriaceae. *Antimicrob. Agents Chemother.* 60(7)**,** 4351-4354. doi: 10.1128/Aac.00550-16.

Liu, Y.Y., Wang, Y., Walsh, T.R., Yi, L.X., Zhang, R., Spencer, J., et al. (2016). Emergence of plasmid-mediated colistin resistance mechanism MCR-1 in animals and human beings in China: a microbiological and molecular biological study. *Lancet Infect Dis* 16(2)**,** 161-168. doi: 10.1016/S1473-3099(15)00424-7.

Weisburg, W.G., Barns, S.M., Pelletier, D.A., and Lane, D.J. (1991). 16S ribosomal DNA amplification for phylogenetic study. *J. Bacteriol.* 173(2)**,** 697-703.

Zheng, B.W., Yu, X., Xu, H., Guo, L.H., Zhang, J., Huang, C., et al. (2017). Complete genome sequencing and genomic characterization of two *Escherichia coli* strains co-producing MCR-1 and NDM-1 from bloodstream infection. *Sci Rep* 7, 17885. doi: 10.1038/s41598-017-18273-2.

Zhi, C.P., Lv, L.C., Yu, L.F., Doi, Y.H., and Liu, J.H. (2016). Dissemination of the *mcr-1* colistin resistance gene. *Lancet Infect Dis* 16(3)**,** 292-293. doi: 10.1016/S1473-3099(16)00063-3.
